# Higher Salaries for Mental Nurses

**Published:** 1918-06-08

**Authors:** 


### Higher Salaries for Mental Nurses.

### THE LONDON COUNCIL PAYS MORE.

Before rising for the Whitsun recess the London County Council sanctioned a new scale of salaries for the female nursing staff of the mental hospitals, viz :—

| Rank | Commencing | Annual Increase | Maximum |
|---|---|---|---|
| | £ | £ | £ |
| Head nurse (day or night) ... ... | 52 (*45*) | 4 (*1*) | 60 (*50–53*) |
| Special charge nurse ... ... ... | 44 (*38*) | 3 (*1*) | 50 (*43*) |
| First-class nurse and night nurse ... | 36 (*31*) | 3 (*1*) | 42 (*37*) |
| Second-class nurse on confirmation after 12 months | 28 (*24*) | 3 (*1*) | 34 (*30*) |
| Probationer nurse for 12 months and until confirmation ... ... ... | 24 (*20*) | — | — |
| Messwoman ... ... ... ... | 36 (*29*) | 3 (*30s.*) | 42 (*37*) |

With board, lodging, washing, and uniform.

(The figures in brackets give the existing rates and show at a glance the proposed increases.)

The scale will secure that at the end of three years' training the nurse will receive such a remuneration as she might expect as a hospital-trained nurse—namely, £80 a year. This sum, for instance, is the commencing rate of pay for school nurses, whose hours of duty are considerably less and whose leave is considerably longer than the hours and leave of a mental hospital nurse.

Under the new scale probationer nurses, instead of starting at £20 a year, will commence at £24, the Council having come to the conclusion that it is advisable to raise the commencing rate in order to attract suitable candidates.

N.B.—The male staff, although boarded, is non-resident; the female staff is all boarded, only a negligible number being non-resident, and the salaries are paid monthly.

☛ For The Economics of Nursing: Viscount Knutsford and "Ierne," see pp. 207-8; College of Nursing Local Centres, p. 216; Matrons' and Sisters' Appointments, p. 216.

